# Intuitive Cell Manipulation Microscope System with Haptic Device for Intracytoplasmic Sperm Injection Simplification

**DOI:** 10.3390/s24020711

**Published:** 2024-01-22

**Authors:** Kazuya Sakamoto, Tadayoshi Aoyama, Masaru Takeuchi, Yasuhisa Hasegawa

**Affiliations:** Department of Micro-Nano Mechanical Science and Engineering, Nagoya University, Nagoya 464-8601, Japan

**Keywords:** macro–micro interaction, macro–micro interface, intuitive micromanipulation system

## Abstract

In recent years, the demand for effective intracytoplasmic sperm injection (ICSI) for the treatment of male infertility has increased. The ICSI operation is complicated as it involves delicate organs and requires a high level of skill. Several cell manipulation systems that do not require such skills have been proposed; notably, several automated methods are available for cell rotation. However, these methods are unfeasible for the delicate ICSI medical procedure because of safety issues. Thus, this study proposes a microscopic system that enables intuitive micropipette manipulation using a haptic device that safely and efficiently performs the entire ICSI procedure. The proposed system switches between field-of-view expansion and three-dimensional image presentation to present images according to the operational stage. In addition, the system enables intuitive pipette manipulation using a haptic device. Experiments were conducted on microbeads instead of oocytes. The results confirmed that the time required for the experimental task was improved by 52.6%, and the injection error was improved by 75.3% compared to those observed in the conventional system.

## 1. Introduction

**Figure 1:**
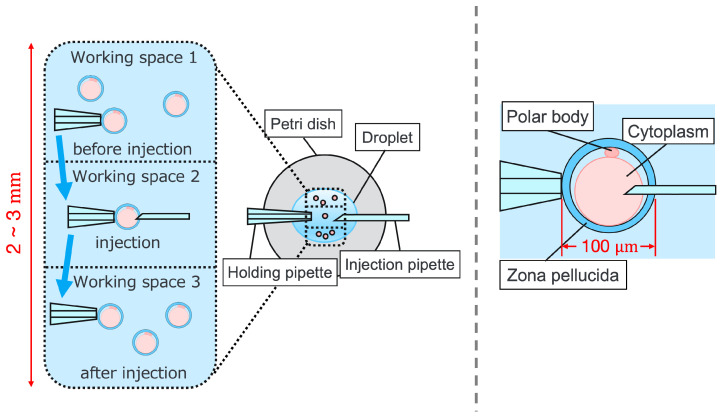
Procedure of ICSI.

Intracytoplasmic sperm injection (ICSI) is the most popular method of insemination worldwide. In the field of assisted reproductive medicine, ICSI is particularly effective for male infertility and has been widely adopted because of its high incidence rate [1,2]. However, ICSI generally requires the direct manipulation of oocytes and sperm under an optical microscope, which is a complicated and delicate operation necessitating high skill. In addition, oocytes are damaged by osmotic pressure during cell manipulation. Therefore, ICSI requires efficient manipulation in a short time. The ICSI procedure is conducted as follows. First, the pre-injected oocyte in workspace 1 in Figure 1 is aspirated using a holding pipette and moved to workspace 2. The cells are then rotated in workspace 2, the polar body is moved to the 12 or 6 o’clock directions, an injection pipette is injected into the cytoplasm of the oocyte, and the sperm are injected. The cells are rotated to avoid damage to the spindles near the polar body. High-resolution images are required to observe the microscopic polar bodies and sperm. The holding pipette is then moved to workspace 3, and the oocyte is ejected [3,4]. The reasons why the ICSI operation is difficult and inefficient are explained from two aspects: the presenting image and the manipulation device. In terms of the presentation image, there is a lack of depth information and an inability to present a wide-area high-resolution image. The rotation and injection of cells requires high-precision work, and the focus position must be adjusted along the depth direction to observe details such as the position of the polar bodies and nuclei of the cells in the high-resolution image. In addition, because cells have individual differences, and the shapes of the cells and polar bodies differ [5], conventional optical microscopes require the operator to change the light intensity and lenses to change the field of view according to the work stage. Observing the three-dimensional trajectories of particles can be challenging due to the limited depth of field of optical microscopes [6]. Certain confocal laser microscopes are capable of three-dimensional (3D) measurements to acquire depth information [7], and several inline holographic microscopes can acquire 3D information of a sample by transmitting light in a noncontact and nondestructive manner. In particular, there are many studies on 3D particle-tracking methods using digital holography [6]. For instance, a clustering-based particle detection method for digital holography has been proposed [8], as well as a method for determining the 3D location of multiple biological targets based on digital holography [9]. However, confocal laser microscopy is not suitable for microinjection owing to the requirement of scanning to measure the depth information and the inability to measure moving cells. Further, inline holographic microscopes are not suitable for microinjection because the conjugate image of the transmitted zero-order light and the object light reduces the resolution of the reproduced image. Although the resolution can be improved using synthetic aperture or pixel super-resolution techniques, multiple images are required; therefore, neither microscope can be introduced to the microinjection process, which requires a spatial resolution of a few micrometers [10,11], a field of view of 3 × 3 mm, and real-time visual feedback. Therefore, we have developed a field-expanding microscope that can capture both a wide field of view and high-resolution images using high-speed viewpoint movements with a galvano-mirror [12,13]. Furthermore, we have developed a microscopy system that uses a field-expanding microscope for the real-time 3D presentation of oocytes and manipulators on a holographic display [14]. However, the operating device is not intuitive because the micromanipulators used to move the pipettes and joysticks used in conventional devices do not correspond to each other in terms of the degrees of freedom (DOFs) of operation. Micromanipulators have 3-DOFs in translation, whereas joysticks have 2-DOFs in translation and 1-DOF in rotation. In addition, the manipulator must switch to an injector during the manipulation to suck and grasp the cells. Although there are examples of biomedical applications using haptic devices, haptic devices are not currently used in ICSI due to the difficulty of the real-time sensing of the three-dimensional position of the oocyte and the contact force during the ICSI process. To simplify these complicated operations, several studies have been conducted to automate cell manipulation. For example, the automation of a simple task, such as picking up and placing small objects, can be achieved by using MEMS micro-grippers with controllable plunge structures [15]. In particular, the automation of cell rotation, which is a particularly important but difficult operation, has been attempted in several ways [4], such as using electric [16], magnetic [17], sound [18,19], and light [20,21] fields. Furthermore, mechanical [22] and fluidic contacts [23,24] have been used to achieve automation. However, although these noncontact rotation methods are suitable for rotating cells at a constant speed, their effects on the developmental process of oocytes have not been evaluated and are risky. Moreover, the light field also has a low power output, which may not be sufficient to rotate the oocytes (>100 μm) or may cause optical damage. Mechanical contact also has the potential for unexpected physical damage [25] because of the difference in the rotation method from the way it is performed in an actual ICSI [22]. In addition, research has been conducted on the automated microinjection of zebrafish as an automated microinjection method [26,27]. Genome editing has a low success rate and requires a large number of trials, and therefore is suitable for automation, where efficiency is more important than accuracy. However, the application of this technique to ICSI is challenging, as this method requires complex manipulations where oocytes and sperm are valuable; moreover, the success rate is an important factor. Automatic ICSI is a complex process requiring the precise control and coordination of various steps. Any error in any of these steps could result in fertilization failure or damage to the gametes. AI models and robotic systems used for automated ICSI are still in their infancy and require further data and training [28]. There are also concerns about the potential for algorithmic bias and the difficulty of real-time error checking [29]. All of these methods and devices differ from conventional manual ICSI, but their effect on cell development rates has not been evaluated. Therefore, we have developed a system that supports human cell manipulation and enables inexperienced operators to perform cell manipulation on par with or better than experienced operators. In this study, in addition to a microscope system that can switch between high-resolution, wide-area imaging and real-time 3D imaging according to the manipulation stage, two intuitive haptic devices that may present a sense of force are used to solve the problem of joysticks as a manipulation device. The haptic devices are used to manipulate the grasping of cells as if they were aspirating or ejecting cells; furthermore, the force sensation is presented to the operator according to the degree of suction, thereby realizing the intuitive suction and ejection of cells. In addition, an automatic position adjustment function during injection and a puncture direction fixation function are used to guide accurate injection. The effectiveness of the proposed system has been experimentally evaluated using porcine embryos to simulate ICSI.

## 2. Intuitive Cell Manipulation Microscope System

### 2.1. System Configuration

The system presents the operator with either an extended-field-of-view image created using the aforementioned galvanometer mirror and ETL or a 3D image created using ETL and a hologram display. The microscopic imaging function, which provides a high resolution with a wide field of view, allows the system to observe all the objects in the droplet without moving the stage.

**Figure 2:**
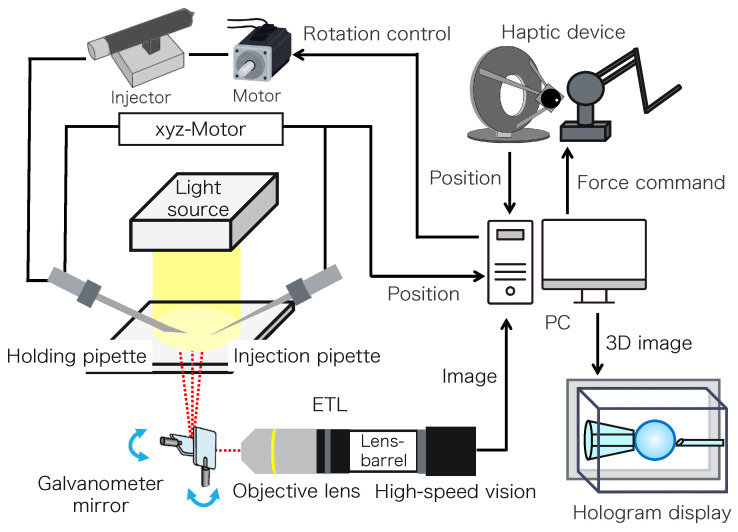
Configuration of the proposed system.

**Figure 3:**
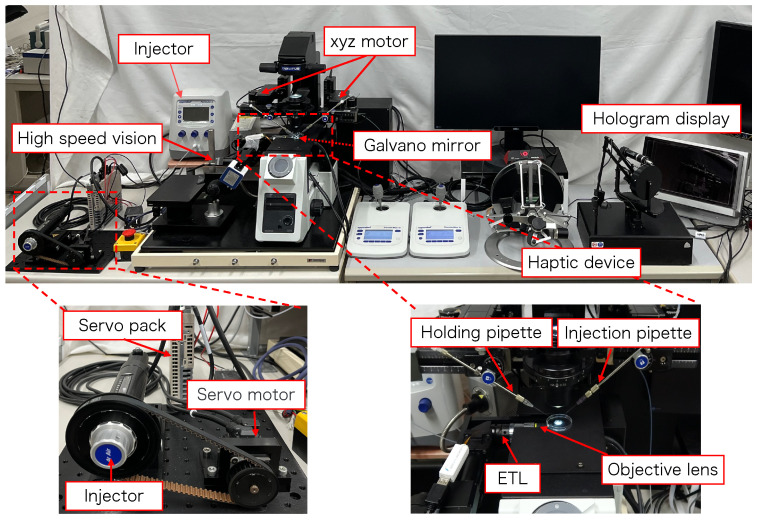
Overview of the proposed system.

Figure 2 shows the configuration of the proposed micromanipulation system and Figure 3 illustrates the system. The system comprises an inverted microscope (IX73, OLYM-PUS), objective lens (LWD95mm, 10X, Mitutoyo, Sakado, Japan), high-speed vision (MQ003MG-CM, Ximea, Lakewood, CA, USA), variable focus lens (EL-10-30-C-VIS-LD-MV, Optotune, Dietikon, Switzerland), lens driver (Lens Driver 4, Optotune, Dietikon, Switzerland), 2-axis galvanometer mirror (6210HSM 6 mm 532 nm, Cambridge Technology, Cambridge, UK), control PC (OS: Windows 10 Home 64bit, CPU: Intel(R) Core(TM) i9-9900KF 3.60 GHz, RAM: 32 GB, GPU: NVIDIA GeForce RTX 2080 SUPER), D/A board (PEX-340416, interface), counter board (PEX-632104, interface), light source (LA-HDF158AS, Hayashirepic Corporation, Tokyo, Japan), joystick-mounted micromanipulator (TransferMan 4r, Eppendorf, Vienna, Austria), microinjector (FemtoJet 4i, Eppendorf, Vienna, Austria), microinjector (CellTram 4r Air, Eppendorf, Vienna, Austria), hologram display (The Looking Glass 15.6 Pro, Looking Glass Factory, New York, NY, USA), haptic device (Phantom Premium 1.5 High Force, 3D Systems, Rock Hill, SC, USA), haptic device (omega.7, Force Dimension, Nyon, Switzerland), servo motor (SGM7A-A5AFA21 Yaskawa, Kitakyushu, Japan), SERVOPACK (SGD7S -R70F00A, Yaskawa, Kitakyushu, Japan), timing belt (HTBN475S5M-100, Misumi, Tokyo, Japan), pulley (HTPB27S5M100-A-P8, HTPB49SM100-B-P41, Misumi, Tokyo, Japan), and optical breadboard (MB2530/M, Thorlabs, Newton, NJ, USA).

### 2.2. Micromanipulation with Haptic Device

The proposed system integrates a field-of-view-expanding microscope using high-speed eye movement with galvano-mirrors, which have a wide field of view and high resolution, and a microscope system [12,13] that displays oocytes and micromanipulators (microscopic manipulation objects) in real time on a holographic display in 3D. We constructed an image presentation system that can switch the presented images according to the operation stage of the microsurgical manipulation by integrating the microscope system [14], which displays oocytes and micromanipulators as real-time 3D images on a holographic display. A simple algorithm for the system is presented in Algorithm 1. *p*, *h*, *e*, and θ denote the position vectors of the pipette, haptic device, oocyte, and injector, respectively. Further, *w*, c1, and c2 denote the position vectors of the world coordinate system, the camera coordinate system of the 3D image, and the camera coordinate system of the field-of-view extended image, respectively. The subscript 0 indicates the initial coordinates and *M* denotes the coordinate transformation matrix. The system converts the world coordinates of the pipette obtained from the encoder to the camera coordinates of the pipette and uses them together with the camera coordinates of the oocyte obtained via circle detection for support. The operator changes the world coordinates of the pipette and injector by operating the haptic device in both the 3D and the extended-field-of-view images. Switching between the 3D image and the extended-field-of-view image is achieved by pressing a switch on the haptic device.
**Algorithm 1** Algorithm of micromanipulation system.  1:$p_{w0},\;h_{w0},\theta_{w0}\leftarrow initial\;vector$  2:$p_{w}\leftarrow p_{w0}$  3:$h_{w}\leftarrow h_{w0}$  4:$\theta_{w}\leftarrow \theta_{w0}$  5:**loop**  6:    Obtain 3D images.  7:    $e_{c1}\leftarrow new\;vector$  8:    The operator moves the pipettes using haptic device.  9:    $h_{w}\leftarrow new\;vector$10:    $p_{w}\leftarrow p_{w0}+M_{hp}\left(h_{w}-h_{w0}\right)$11:    $p_{c1}\leftarrow M_{wc1}p_{w}$12:    $\theta_{w}\leftarrow \theta_{w0}+M_{h\theta }\left(h_{w}-h_{w0}\right)$13:**end loop**14:The operator switches the presented image with a button.15:**loop**16:    Obtain expanded images.17:    $e_{c2}\leftarrow new\;vector$18:    Operator moves the pipettes using haptic device.19:    $h_{w}\leftarrow new\;vector$20:    $p_{w}\leftarrow p_{w0}+M_{hp}\left(h_{w}-h_{w0}\right)$21:    $p_{c2}\leftarrow M_{wc2}p_{w}$22:    $\theta_{w}\leftarrow \theta_{w0}+M_{h\theta }\left(h_{w}-h_{w0}\right)$23:**end loop**

## 3. Intuitive Pipette Manipulation with Haptic Device

### 3.1. Operation Interface

The system uses two haptic devices instead of conventional joysticks and injectors as the operating interface. A 7-DOFs haptic device (omega.7) with a grasping function is used for holding the pipette manipulation, and a pen-type haptic device (Phantom Premium 1.5 High Force) is used for injection pipette manipulation, aiming at intuitive pipette manipulation. The translational movement of each pipette is controlled by converting the position coordinates of the haptic device to the position of the corresponding micropipette and controlling the micromanipulator.

### 3.2. Cell Suction/Discharge

**Figure 4:**
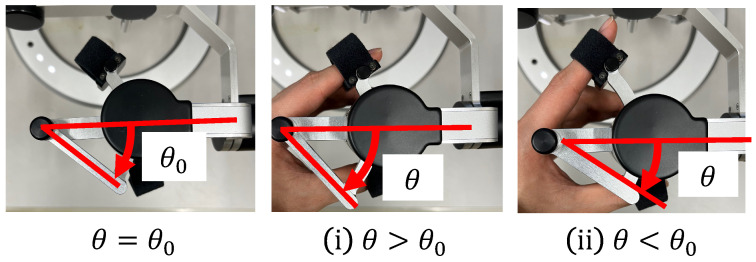
Grip angle of the manipulation interface.

**Figure 5:**
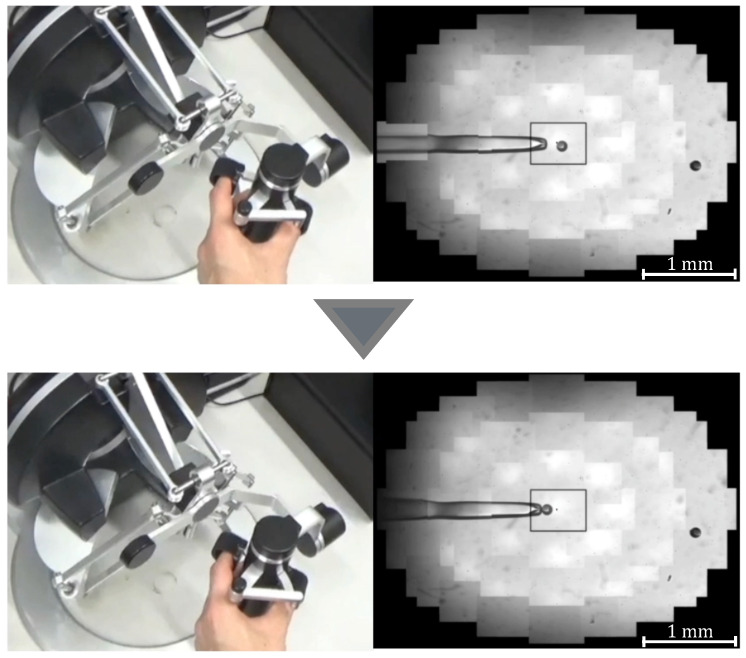
Manipulation of a target with suction pressure.

**Figure 6:**
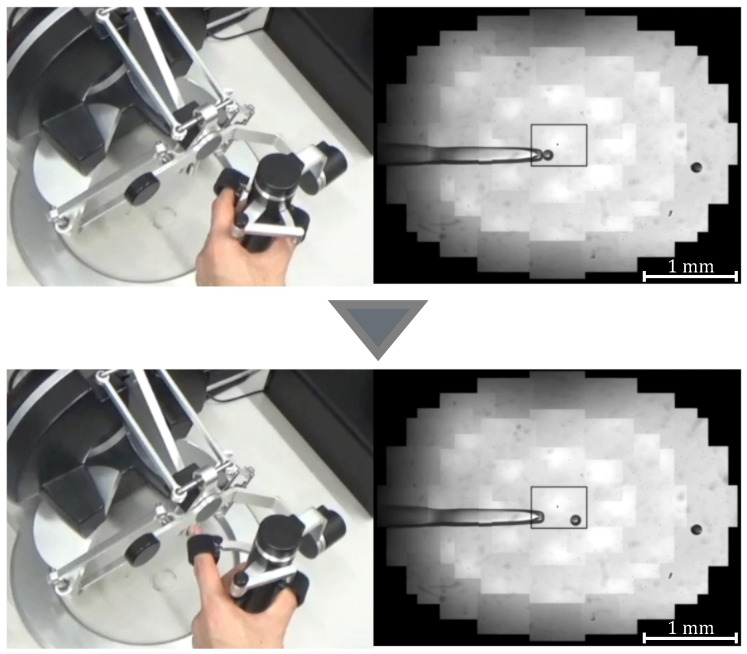
Manipulation of a target with discharge pressure.

In conventional systems, the cells are aspirated and discharged by rotating the injector and applying positive or negative pressure to the holding pipette. Conventionally, this operation requires the user to change the joystick and injector during the operation, thus rendering the operation cumbersome. In the proposed system, one DOF for grasping the haptic device with a grasping function used to manipulate the holding pipette is allocated to the suction and dispensing of cells. Therefore, the proposed system enables intuitive and efficient manipulation. The rotation of the injector is controlled by a motor that is connected to it via a timing belt. This decision was made because the belt is quiet and easy to maintain. When the haptic device is grasped, the motor rotates $N_{m}$ in Equation (1) according to the grasping angle θ of the haptic device, as shown in Figure 4; then, the injector rotates $N_{i}$ in Equation (2) through the timing belt.

(1) $$\begin{array}{cc} N_{m} & =\frac{G}{n}\left(\theta -\theta_{0}\right), \end{array}$$

(2) $$\begin{array}{cc} N_{i} & =\frac{1}{n}\left(\theta -\theta_{0}\right), \end{array}$$

where *G* denotes the gear ratio, *n* is a constant, and $\theta_{0}$ is the initial grasp angle of the haptic device. When $\theta >\theta_{0}$, the injector rotates counterclockwise and negative pressure is applied to the tip of the holding pipette to perform the suction operation; when $\theta <\theta_{0}$, the injector rotates clockwise and positive pressure is applied to perform the discharge operation. Figure 5 and Figure 6 show the manner in which the cells are aspirated and discharged using these injectors. The grasping of the haptic device enables the suction and discharge of the cells.

## 4. Manipulation Assistance with Force Presentation

### 4.1. Holding Pipette

**Figure 7:**
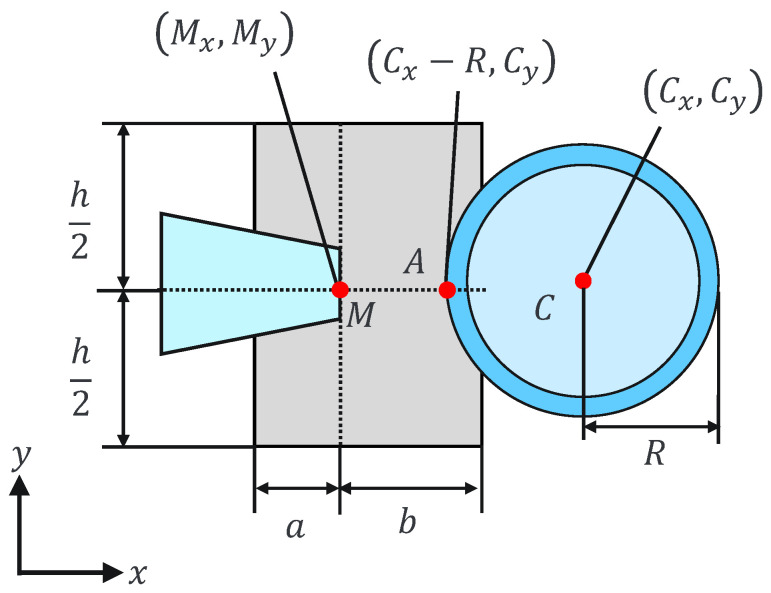
Contact situation.

**Figure 8:**
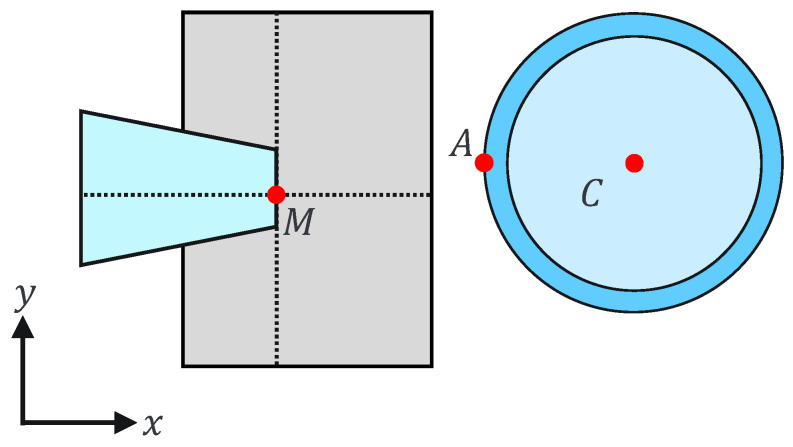
Noncontact situation.

In this system, several assist functions are implemented to improve the operability and efficiency of the manipulation using a haptic device. For the holding pipette manipulation, we have implemented a function that applies the force F to the grasping part of the haptic device, depending on the grasping angle of the device and the contact between the cell and the holding pipette:

(3) $$\begin{array}{c} F=\left\{\begin{array}{cc} -k_{1}\left(\theta -\theta_{0}\right)e & \left(\mathrm{Without}\;\mathrm{contact}\right)\\ -k_{2}\left(\theta -\theta_{0}\right)e & (\mathrm{With}\;\mathrm{contact}), \end{array}\right. \end{array}$$

where $k_{1}$ and $k_{2}$ are the scale factors ($k_{1}<k_{2}$) for the cell and holding pipette with noncontact and contact, respectively, and e is the unit vector in the grasping direction of the haptic device. The contact judgment is made using the coordinates of the holding pipette, grasping angle, and circle detection of the cell. The holding pipette coordinates, grasping angle, and cell circle detection are determined through image processing, as outlined in [14]. The acquisition of the pipette coordinates obtained from the coordinate transformation has a delay of approximately several milliseconds and may deviate from the actual pipette coordinates. Therefore, a contact judgment method that considers slight deviations in the pipette position must be developed. Specifically, when the following inequality is satisfied, the holding pipette is considered to be in contact with the cell.

(4) $$\begin{array}{c} \left\{\begin{array}{c} M_{x}-a\leq C_{x}-R\leq M_{x}+b\\ M_{y}-\frac{h}{2}\leq C_{y}\leq M_{y}+\frac{h}{2}, \end{array}\right. \end{array}$$

where $M_{x}$ and $M_{y}$ are the holding pipette tip coordinates and *a*, *b*, and *h* are constants. This can be explained geometrically as shown in Figure 7 and Figure 8. The holding pipette is considered to be in contact with the cells, as shown in Figure 7. Conversely, when the holding pipette tip is not included in the gray region, as shown in Figure 8, it is judged to be noncontact.

This assist function is used to remind the operator of the positive or negative pressure applied to the holding pipette by presenting a force to the operator along the direction corresponding to the suction or discharge, with a magnitude proportional to the positive or negative pressure applied by the injector. By changing the scale factor between contact and noncontact, the operator can intuitively judge the presence or absence of contact based on a sense of force.

### 4.2. Injection Pipette

**Figure 9:**
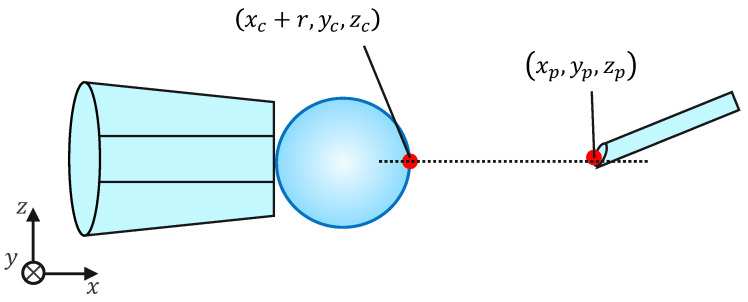
Injection with a guide function.

The injection pipette operation is equipped with a guide function. This guide function comprises two parts: automatic position adjustment during injection and puncture direction fixation. Figure 9 shows the concept of the guiding function during injection.

1. Automatic Positioning
  When the guided mode is selected, the *y* and *z* coordinates of the injection pipette tip are automatically aligned with the *y* and *z* coordinates of the cell, respectively, enabling puncturing at the exact position without manual alignment.
  (5) $$\begin{array}{c} y_{p}=y_{c} \end{array}$$
  (6) $$\begin{array}{c} z_{p}=z_{c} \end{array}$$
2. Fixation of puncture direction
  After automatic position adjustment in the guide mode, only operations along the *x*-axis direction are accepted to prevent misalignment of the puncture direction.

## 5. Experiment

### 5.1. Method

**Figure 10:**
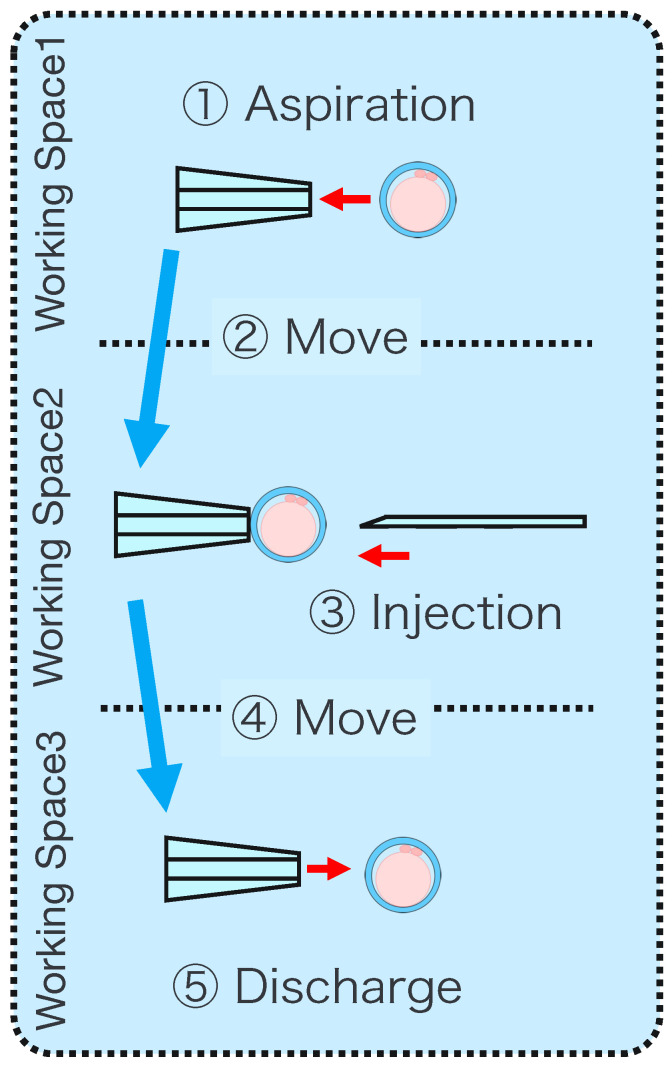
Overview of the experimental procedure.

A subject experiment was conducted to verify the effectiveness of the proposed system. The purpose of our proposed system is to facilitate cell transport with the holding pipette and positioning during injection including the depth direction by using the injection pipette. Therefore, in this experiment, the cell transport and positioning during injection are set as the evaluation tasks. The experimental conditions are given in Table 1. A schematic of the experiment is presented in Figure 10, and the details of the experimental task are presented below. Six sets of this task were performed in each condition by six adults who had no experience with fine manipulation for at least one week. The manipulation using the haptic device included the aforementioned assistance. This study was approved by the Ethics Committee of the Faculty of Engineering at Nagoya University (20–23). Informed consent was obtained from all subjects.

1. Aspirate the microbeads (diameter 100±1.5μm) in Working space 1 with a Holdin pipette while viewing the extended view image.
2. Move to Working space 2 and switch to a 3D image.
3. Contact the tip of the injection pipette with the *x*-coordinate end of the microbeads.
4. Switch to the extended view image and move the microbeads to Working space 3.
5. Eject the microbeads from the holding pipette.

**Figure 11:**
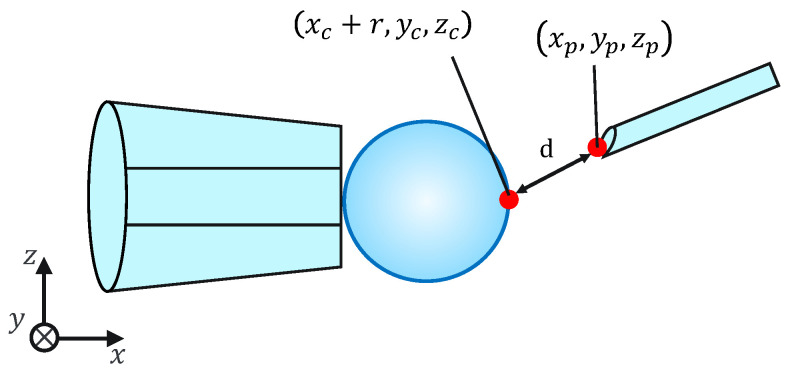
Injection error ‘*d*’.

Because this evaluation task is not affected by the mechanical properties of the cells, for simplicity, microbeads are used instead of cells. To evaluate the injection accuracy, we measured the distance *d* between the microbeads, *x* coordinates of the largest point, and injection pipette tip coordinates, as shown in Figure 11; subsequently, we evaluated whether the injection pipette could be moved to the target point. The distance *d* is given by the following equation:

(7) $$\begin{array}{c} d=\sqrt{{\left(x_{c}+r-x_{p}\right)}^{2}+{\left(y_{c}-y_{p}\right)}^{2}+{\left(z_{c}-z_{p}\right)}^{2}}. \end{array}$$

**Figure 12:**
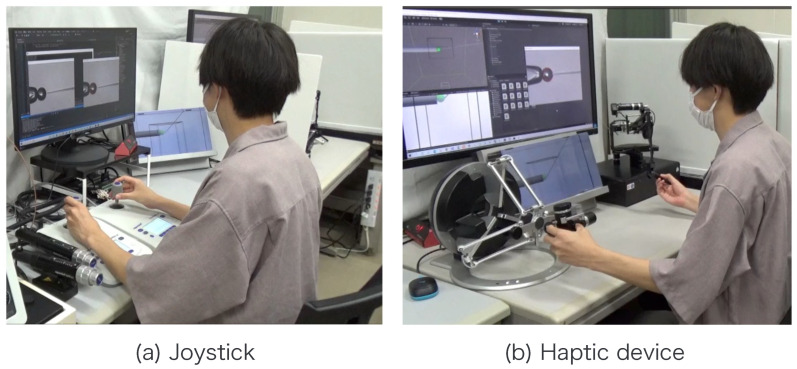
Experimental scene.

Note that $x_{c}$, $y_{c}$, $z_{c}$, $x_{p}$, $y_{p}$, $z_{p}$, and *r* are obtained from the image processing and the movement of the micromanipulator based on the work [14]. The switching of the presentation image was performed via the keypad. The images were switched using a keyboard operation or by pressing a physical button on the haptic device used to control the injection pipette. The experimental setup is shown in Figure 12.

### 5.2. Results

**Figure 13:**
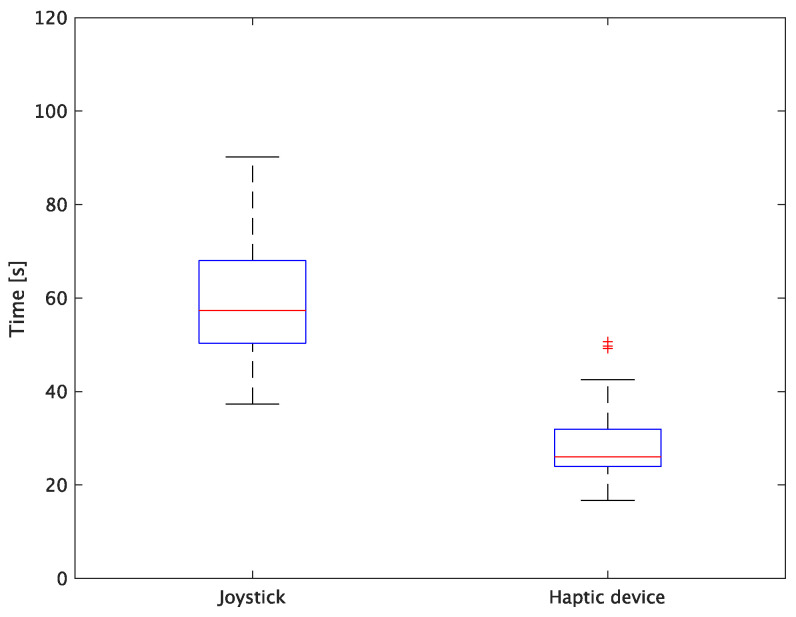
Box plot of task time.

**Figure 14:**
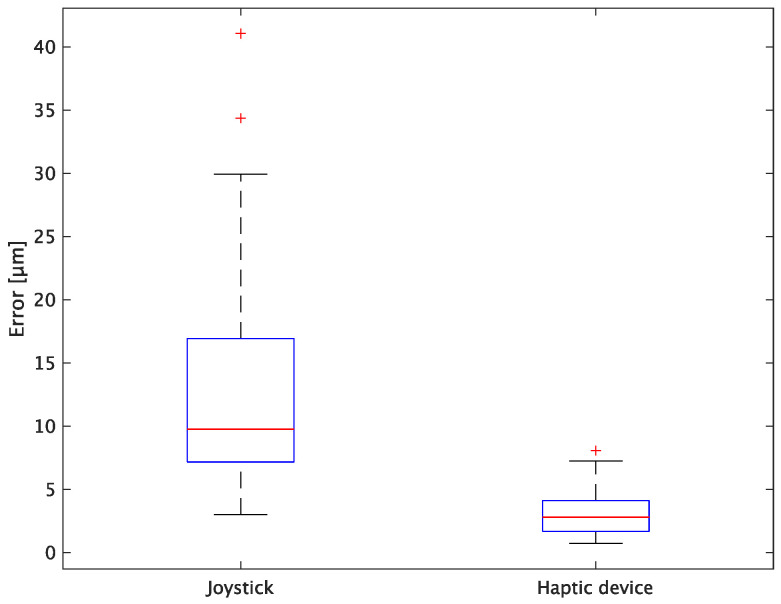
Box plot of total error.

**Figure 15:**
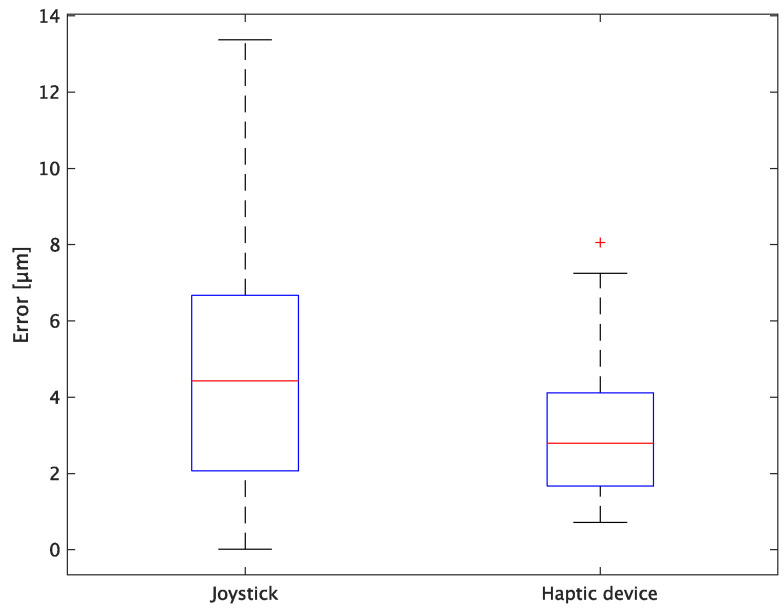
Box plot of x-axis error.

**Figure 16:**
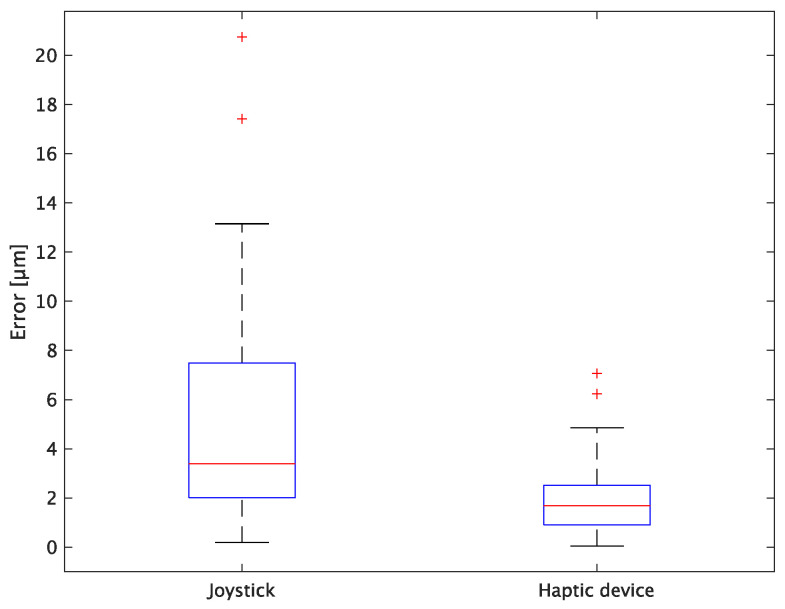
Box plot of y-axis error.

**Figure 17:**
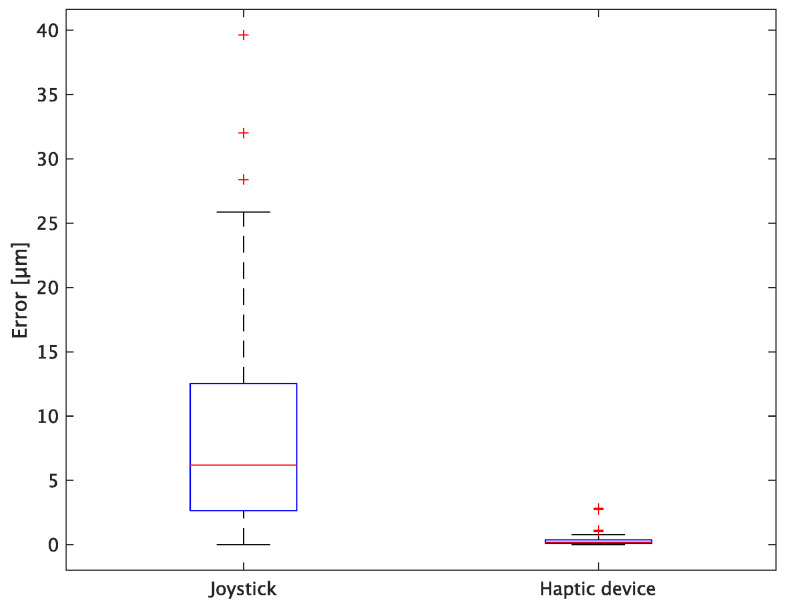
Box plot of z-axis error.

The experimental results are as follows: Table 2 presents the average task performance times under conditions (a) and (b) for each subject, Table 3 presents the error during the injection, and Table 4, Table 5 and Table 6 show the average errors along the *x*, *y*, and *z* axes, respectively. Further, Table 3, Table 4, Table 5 and Table 6 show the average errors along the *x*, *y*, and *z* axes, respectively. Figure 13 shows a box-and-whisker diagram of the task execution time. Moreover, Figure 14 shows a box-and-whisker diagram of the error, and Figure 15, Figure 16 and Figure 17 show the box-and-whisker diagrams of the errors along the *x*, *y*, and *z* axes, respectively. The *x*, *y*, and *z* axes represent box plots of the error in each direction.

### 5.3. Discussion

The experiment comprised a two-condition task for six subjects. Because the distribution of the results was not expected to be normal, a nonparametric Wilcoxon signed-rank test was used. The test results showed that the *p* values were significantly different for the task performance time and accuracy. The task performance time was reduced by an average of 52.6% by changing the control device from a joystick to a haptic device. The largest decrease was observed in C at 62.0% and the smallest decrease was observed in E at 43.2%. The task performance time decreased significantly regardless of the subject. The significant decrease in the task performance time can be attributed to the change from the joystick to the haptic device for both pipette manipulations.

First, the time required to pick and place the microbeads was significantly reduced by changing the holding pipette manipulation device from a joystick to a haptic device. This can be attributed to the fact that the proposed system can aspirate and dispense cells by grasping the haptic device without changing the joystick to the injector, whereas the conventional system requires the user to change the joystick to the injector when aspirating and discharging cells. In addition, by changing the control device of the injection pipette to a haptic device, the positioning during injection was implemented as an assist function. After the operator moved the holding pipette to the center of Workspace 2, the cells (microbeads) and the injection pipette were automatically aligned, thereby enabling a significant reduction in time.

In terms of accuracy, the error *d* was reduced by an average of 75.3% by changing the operating device from a joystick to a haptic device. The largest percentage decrease was 85.9% for D, and the smallest percentage decrease was 54.7% for A. Those with a larger percentage decrease in the task time tended to exhibit a smaller percentage decrease in errors, whereas those with a smaller percentage decrease in task time tended to exhibit a larger percentage decrease in error. This smaller error is attributed to the manipulation device of the injection pipette. The injection pipette has an assist function that fixes the direction of injection, allowing the user to concentrate only on the *x* axis after alignment, thus enabling a highly accurate injection. Furthermore, by checking the errors along the *x*, *y*, and *z* axes, the *x* component of the error *d* was reduced by an average of 61.7%. The largest decrease was observed in A (81.6%), and the smallest in B (20.2%). The *y* component decreased by an average of 59.6%. The *y* component decreased by an average of 59.6%, with F having the largest decrease of 81.6% and A having the smallest decrease, increasing the error by 50.5%. The *z* component decreased by an average of 96.0%. The largest decrease was observed in B (98.7%), and the smallest decrease was observed in F (91.3%). The error in the *x* axis is considered to be smaller because the subject can concentrate on the *x*-axis manipulation, as described above, even in the absence of a guiding function. Owing to the lack of a guide function, the rate of reduction varied significantly from subject to subject. The *y* and *z* axes were improved using the guide function. The *z* axis was more difficult to confirm visually than the *x* and *y* axes’ coordinates simultaneously. The guide function allows the injection pipette to be aligned with the cell in the same line. However, there is a slight delay between the command and the actual movement of the pipette tip. During this delay, the position of the cell changes slightly, causing the error in the *y* axis and *z* axis to deviate from zero. The haptic device is a three-dimensional manipulation interface. However, excessive degrees of freedom can reduce usability depending on the task. For instance, it may be necessary to limit the movement of one axis when working on a two-dimensional plane. Nevertheless, the proposed system was effective in simplifying the manipulation of ICSI because the task performance time and error were clearly reduced using the proposed system in all subjects.

## 6. Demonstration

**Figure 18:**
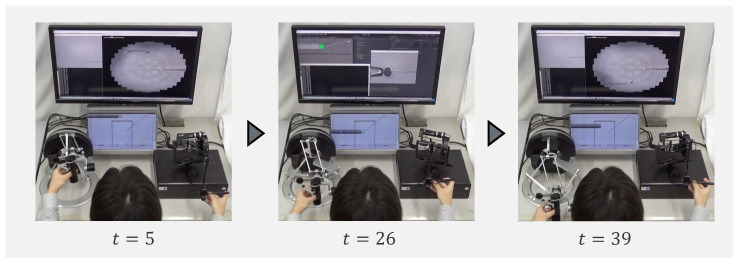
Overhead view of the operation during the demonstration.

**Figure 19:**
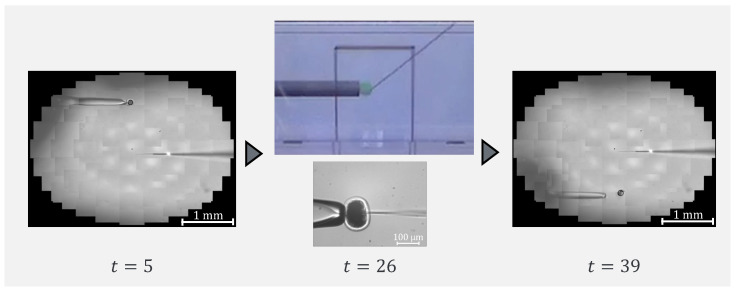
Presented image during demonstration.

**Figure 20:**
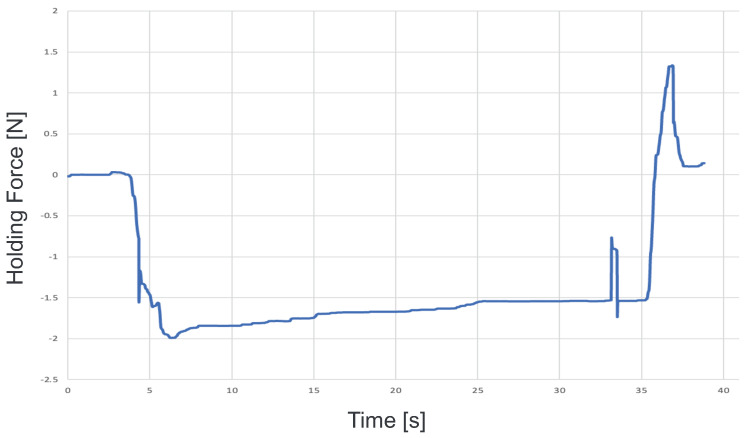
Force graph presented during the demonstration.

To confirm that our proposed system can be applied to actual cells, a demonstration was performed using porcine embryos. An overhead view of the operation during the demonstration is shown in Figure 18, and the displayed 2D or 3D images are shown in Figure 19. Figure 20 shows the generated force from the haptic device during the demonstration. Although the manipulation is almost the same as in the experiment using microbeads, in this demonstration the cells are actually punctured. It was confirmed that the proposed system can be used for ICSI procedures of transporting and puncturing cells.

## 7. Conclusions

In this study, we proposed an intuitive cell manipulation microscope system with haptic devices for ICSI simplification. The proposed system is capable of switching between high-resolution, wide-area images and 3D images in real time. In addition, we used two intuitive haptic devices that can present a sense of force, thereby achieving high efficiency in cell suction and ejection operations and high precision in injection operations. The effectiveness of the proposed system was confirmed through manipulation experiments on microbeads that mimic the ICSI operation and demonstration on porcine embryos. This study shows the potential advantages of systems that assist human operators in performing the ICSI procedure rather than fully automating it. Such assistive systems could be particularly valuable in ICSI, where success rates are critical. In the future, the effectiveness of the proposed system will be demonstrated by comparing the developmental processes of embryos. During ICSI, in addition to pick-and-place and positioning, the procedure involves other challenging operations. One of these crucial tasks is cell rotation, which must be performed carefully to prevent damage to the oocytes. The application of our system to support the cell rotation task is left for future study.

## Figures and Tables

**Table 1:** Experimental conditions.

Condition	Operation Interface
(a)	Joystick
(b)	Haptic device

**Table 2:** Mean time of task performance (s).

Subjects	Joystick	Haptic Device
A	64.4	28.1
B	64.8	29.6
C	50.0	19.0
D	63.5	34.9
E	56.9	32.3
F	54.9	24.1
avg.	59.1	28.0

**Table 3:** Mean total error (μm).

Subjects	Joystick	Haptic Device
A	7.13	3.22
B	10.91	2.09
C	10.05	4.46
D	17.33	2.44
E	15.42	3.08
F	15.98	3.63
avg.	12.80	3.16

**Table 4:** Mean error of x axis (μm).

Subjects	Joystick	Haptic Device
A	5.20	0.96
B	1.84	1.47
C	5.06	2.96
D	3.72	1.42
E	7.00	1.52
F	6.30	2.83
avg.	4.85	1.86

**Table 5:** Mean error of y axis (μm).

Subjects	Joystick	Haptic Device
A	1.90	2.86
B	2.15	1.10
C	6.64	2.74
D	5.25	1.67
E	7.50	2.45
F	6.20	1.14
avg.	4.94	2.00

**Table 6:** Mean error of z axis (μm).

Subjects	Joystick	Haptic Device
A	3.49	0.23
B	10.15	0.14
C	3.66	0.26
D	15.23	0.27
E	10.13	0.27
F	11.32	0.98
avg.	9.00	0.36

## Data Availability

Data are contained within the article and Supplementary Materials.

## Supplementary Materials

The following supporting information can be downloaded at: https://www.mdpi.com/article/10.3390/s24020711/s1, Video S1: Demonstration of cell manipulation using a microscope system with haptic device.
